# Quantitative Proteomics of the Infectious and Replicative Forms of *Chlamydia trachomatis*

**DOI:** 10.1371/journal.pone.0149011

**Published:** 2016-02-12

**Authors:** Paul J. S. Skipp, Chris Hughes, Thérèse McKenna, Richard Edwards, James Langridge, Nicholas R. Thomson, Ian N. Clarke

**Affiliations:** 1 Centre for Proteomic Research, Centre for Biological Sciences, University of Southampton, Southampton, United Kingdom; 2 Waters Corporation, Stamford Avenue, Wilmslow, United Kingdom; 3 Pathogen Genomics, The Wellcome Trust Sanger Institute, Hinxton, United Kingdom; 4 Molecular Microbiology Group, School of Medicine, University of Southampton, Southampton, United Kingdom; 5 School of Biotechnology and Biomolecular Sciences, The University of New South Wales, Australia, and School of Biological Sciences, University of Southampton, Southampton, United Kingdom; University of Würzburg, GERMANY

## Abstract

The obligate intracellular developmental cycle of *Chlamydia trachomatis* presents significant challenges in defining its proteome. In this study we have applied quantitative proteomics to both the intracellular reticulate body (RB) and the extracellular elementary body (EB) from *C*. *trachomatis*. We used *C*. *trachomatis* L2 as a model chlamydial isolate for our study since it has a high infectivity:particle ratio and there is an excellent quality genome sequence. EBs and RBs (>99% pure) were quantified by chromosomal and plasmid copy number using PCR, from which the concentrations of chlamydial proteins per bacterial cell/genome were determined. RBs harvested at 15h post infection (PI) were purified by three successive rounds of gradient centrifugation. This is the earliest possible time to obtain purified RBs, free from host cell components in quantity, within the constraints of the technology. EBs were purified at 48h PI. We then used two-dimensional reverse phase UPLC to fractionate RB or EB peptides before mass spectroscopic analysis, providing absolute amount estimates of chlamydial proteins. The ability to express the data as molecules per cell gave ranking in both abundance and energy requirements for synthesis, allowing meaningful identification of rate-limiting components. The study assigned 562 proteins with high confidence and provided absolute estimates of protein concentration for 489 proteins. Interestingly, the data showed an increase in TTS capacity at 15h PI. Most of the enzymes involved in peptidoglycan biosynthesis were detected along with high levels of muramidase (in EBs) suggesting breakdown of peptidoglycan occurs in the non-dividing form of the microorganism. All the genome-encoded enzymes for glycolysis, pentose phosphate pathway and tricarboxylic acid cycle were identified and quantified; these data supported the observation that the EB is metabolically active. The availability of detailed, accurate quantitative proteomic data will be invaluable for investigations into gene regulation and function.

## Introduction

*Chlamydia trachomatis* is the commonest cause of bacterial sexually transmitted infection in Europe and the USA [1]. *C*. *trachomatis* genital tract infections are frequently asymptomatic and thus if treatment is not sought the infection is spread silently [2, 3]. The highest rates of infection are amongst the 16–24 year old age group [4, 5]. Symptomless, and therefore untreated, *C*. *trachomatis* infections can give rise to severe, long term sequelae. In women, complications of chronic chlamydial infections are pelvic inflammatory disease, tubal infertility and ectopic pregnancy [6, 7]. *C*. *trachomatis* infections are treatable with antibiotics, although treatment failures appear common [8]. The cases of *C*. *trachomatis* continue to rise despite significant efforts to control the spread of infection. The patterns of chlamydial infection have shown some significant increasing trends in recent years with the emergence of a new variant strain in Sweden and an epidemic of lymphogranuloma venereum in the MSM population[9, 10]. Thus understanding the structure and function of the infectious (elementary body or EB) and dividing (reticulate body or RB) forms of *C*. *trachomatis* is of fundamental importance to developing better treatments and new diagnostic procedures.

*C*. *trachomatis* is an obligate intracellular pathogen; eukaryotic host cell infection begins when the extracellular infectious form of the microorganism, the EB binds to a susceptible cell [11]. The bacteria are taken up within a cytoplasmic vesicle, which is rapidly subverted to become a protected, membrane-surrounded environment known as an inclusion within which the chlamydia can grow and divide. The first steps in this process involve the re-organisation of the EB into a RB within an inclusion, and takes some 8–18 hours depending on the host cell and chlamydial strain. RBs divide by binary fission and the first-formed RB will undergo seven to eight binary fissions before the progeny re-condense to form EBs, which are subsequently released by host cell lysis [12]. A productive round of chlamydial infection, known as a developmental cycle, concludes with the release of some 200–500 infectious progeny per inclusion, as is the case with the model strain of *C*.*trachomatis* L2 used in this study to infect BGMK cells [13]

A key challenge in chlamydial biology is to define the differences at the molecular level between EBs and RBs. These have involved either transcriptional [14, 15] and proteomic approaches[16, 17]. It is difficult to infer levels of protein expression and stages of the developmental cycle from transcriptional data alone due to the influence of post-transcription regulatory processes, thus we have proposed that evaluation of the developmental cycle should be standardised by measuring chromosomal and plasmid replication rates [12]. A further confounding factor is defining what constitutes an RB, especially as the co-ordination of the developmental cycle rapidly looses synchrony once RB division begins [14]. In this respect, we have purified RBs from just before chromosomal replication, and hence RB division begins, these are the first-formed RBs. In addition, we have minimised the possibility of multiply infected and hence metabolically stressed host cells by a using a multiplicity of infection (MOI = 3).

Recently, it has been shown that EBs are metabolically active, this is a paradigm shift in our understanding of the basic physiology of Chlamydia [18]. The accurate direct measurement of protein levels and comparison between defined populations of EBs and RBs, extracted directly from their natural environment, will provide new insights into the active processes within EBs and RBs.

We have employed two-dimensional reverse phase UPLC to fractionate chlamydial peptides prior to MS^E^ analysis providing absolute amount estimates of the identified proteins, offering a number of significant advantages over relative quantification. For example, by expressing data in terms of molecules per cell, proteins can be ranked in terms of their abundance and measures of the amount of energy invested in their synthesis calculated, facilitating the identification of potentially rate-limiting components. Such data is a prerequisite for building quantitative predictive models of cellular behavior and assists inter-study comparisons as each measurement is independent and does not have to be linked to an equivalent one in a reference sample.

Using an 11-step high-pH, low pH, two stage fractionation, we have achieved extended proteome and protein coverage compared to other reported studies. The data reveal hitherto uncharacterized proteins to be amongst the most abundant components of both the EB and RB forms of *C*. *trachomatis*, and indicate where *C*. *trachomatis* invests its energy in protein synthesis. They also pinpoint proteins with exquisite accuracy that are differentially expressed between EBs and first-formed RBs and hence associated with differences in metabolism, infectivity and survival between these two forms of the pathogen. In particular, it has allowed quantitative characterisation of those proteins involved in energy metabolism providing further information on the metabolic activity profiles within chlamydia during the developmental cycle.

## Materials and Methods

### Chlamydia and cells

*C*. *trachomatis* L2/434/Bu (VR902B) was originally obtained from the ATCC. This isolate was plaqued purified three times and verified by sequence analysis of the *ompA* gene as previously described [16]. *C*. *trachomatis* L2 434/Bu was grown in Buffalo green monkey cells (BGMK) in DMEM supplemented with 10% foetal calf serum containing cycloheximide at 1μg/ml [12]. Stocks of *C*. *trachomatis* were prepared as described previously [19]. The infectivity of EB preparations was titred by serial dilution in 96-well trays. Briefly, mature inclusions were stained using an in-house monoclonal genus-specific antibody that recognizes the chlamydial LPS, the bound monoclonal antibody was detected with a rabbit polyclonal IgG conjugated to β-galactosidase. Specific details of the procedure for staining and counting inclusions are as described [20]. *C*. *trachomatis* and BGMK cells were routinely tested for mycoplasma contamination by fluorescence microscopy using Hoechst No. 33258 staining and using VenorGem® Mycoplasma PCR Detection Kit (MinervaBiolabs, Berlin, Germany).

### Purification of EBs and RBs and quantification of EBs

For large scale purification of EBs and RBs eighteen T-175 tissue culture flasks were infected with a carefully titred inoculum of EBs (moi = 3.0) The earliest stage RBs were purified at 15hrs post infection, some 3–5 hrs before inclusions become visible (in this system) by phase contrast microscopy [12]. EBs were harvested at 48hrs post infection when the proportion of mature EBs were at their maximum and before inclusions started to lyse [12]. Chlamydia–infected BGMK cells were detached from the plastic flasks with PBS containing 0.125% trypsin/0.02% EDTA and then pelleted in DMEM containing 10% FCS at 3,000 x g for 10 mins. The chlamydia-infected BGMK cell pellet was suspended in PBS:H_2_O (1:10), the cells were lysed and EBs and RBs released using a Dounce homogenizer. The cell debris was sedimented at 250 x g for 5 mins and the supernatant retained and mixed with an equal volume of PBS. RBs and EBs were then purified through two cycles of density gradient purification as previously described in detail [16]. Finally, the material was purified on discontinuous urografin gradients with RBs banding at the 34/44% interface and EBs banded at 44/54% interface. RB and EB fractions were collected by sedimentation at 3500 x *g* in a Beckman 55.2 rotor as previously described [16]. These highly purified EBs and RBs were re-suspended in PBS and stored in aliquots at -80°C.

### Genome quantification of RBs and EBs by real-time qPCR

A single copy of the *omcB* gene is located on the *C*.*trachomatis* L2/434/Bu chromosome [19]. The absolute number of genomes in the highly purified RB and EB preparations was accurately determined by performing 5′-exonuclease (TaqMan) assays with unlabelled primers and carboxyfluorescein/carboxytetramethylrhodamine (FAM/TAMRA) dual-labelled probes based on the *omcB* gene as previously described [21]. Briefly, DNA was extracted from 5ul of purified EBs/RBs using the protocol described in Salim et al 2008 [22]. 5ul of this DNA preparation was added to a 20 μl reaction mixture containing forward primer (300 nM), reverse primer (300 nM), probe (100 nM) and TaqMan Universal PCR Master Mix (Applied Biosystems). Real-time PCR cycles were performed in an ABI PRISM 7700 Sequence Detection System (Applied Biosystems) according to the manufacturer’s instructions. This allowed the concentration of EB and RB genomes to be determined per ml of pellet suspension, providing a suitable method of normalizing the samples.

### Expression of CTL0847 in *E*.*coli*, polyclonal sera and immunoblotting

The coding sequence for gene CTL 0847 from *C*.*trachomatis* L2/434/Bu was cloned and expressed using the Xpress™ system (Invitrogen life technologies) which allows expression of a recombinant protein fused at the N–terminual to a six-histidine tag, facilitating purification. CTL0847 specific primer pair, CTL0847_BamHI_F:5’-GGTGGT**GGATCC**ATGACGACGAAACCCAAAAC-3’ and CTL0847_HindIII_R:5’-GGTGGT**AAGCTT**TTACACAGATTTCGTTAATTC-3’ (engineered restriction sites, (*Bam*H1 and *Hind* III) are shown in bold, preceding the complementary recognition sequence) were used to amplify the CTL 0847 gene using *C*.*trachomatis* L2/434/Bu genome as template in a PCR reaction containing Phusion Flash High-Fidelity PCR Master Mix (Thermo Scientific)over 35 cycles of 98°C for 2s, 56°C for 5 s and 72°C for 30 s. The 552 bp amplicons were gel-purified, cleaved with *Bam*H1 and *Hind* III and cloned into pRSETA (Invitrogen). Soluble purified CTL0847 protein, purified using the Xpress system (Invitrogen) according to the manufacturer’s instructions was used to raise a mono specific polyclonal mouse serum as previously described. SDS PAGE and immunoblotting was performed as previously described [23].

### Sample preparation for MS

Highly purified EBs and RBs were pelleted by centrifugation at 13,000 x *g* for 10 min at 4°C and the supernatant discarded. 200 μL of 0.5M triethylammonium bicarbonate (Sigma Aldrich, Poole, Dorset.) containing 10 mM DTT and 0.1% SDS were added to the cells and incubated on ice for 2 h. Bacterial cells were mechanically disrupted using a FastPrep system (Savant) in combination with Lysing Matrix D ceramic beads (Q-Biogene) for 6 cycles, followed each time by incubation on ice. The cell lysate was further processed using 3 cycles of sonication using a Misonix sonicator and microprobe, followed by 1 min incubations on ice. The bacterial lysates were centrifuged at 13,000 x *g* for 10 min to remove cell debris. Protein concentration was determined using the Bradford protein assay (BioRad Laboratories, Hercules, CA, USA). Samples were flash frozen and stored at -80°C until ready for use

### Preparation of protein digests

Total protein lysates of EB and RB preparations were reduced for 1 h at 56°C using 50 mM tris-(2-carboxyethyl) phosphine, alkylated with 200 mM methyl methane-thiosulfonate for 10 min at RT and proteolytically digested using trypsin by the addition of 1:25 (w/w) trypsin-to-protein and incubating overnight at 37°C. 100 μg of the peptide lysates were lyophilized *in vacuo* and re-suspended in 100 mM ammonium formate containing the internal reference protein alcohol dehydrogenase (ADH) at a final concentration of 20 fmol/μl.

### 2D-RPLC-MS^E^

Two-dimensional separations were performed using a nanoAcquity 2D UPLC system (Waters). For the first dimension separation, 4.5 μl of the prepared protein lysates containing 90 fmol of an ADH digest were injected onto a 5μm Xbridge BEH130 C18, 300μm ID x 50mm (Waters) column equilibrated in 20 mM ammonium formate, pH 10 (buffer A). The first dimension separation was achieved by increasing the concentration of acetonitrile (buffer B) in 11 steps consisting of 8.2%, 11.7%, 13%, 14.5%, 15.9%, 17.4%, 18.9%, 20.8%, 23.6%, 45%, 65%. At each step the programmed percentage composition was held for 1 min at a flow rate of 2μl/min and the eluent diluted by buffer C (H_2_O + 0.1% formic acid) from the second dimension pump at a flow rate of 20ul/min, effectively diluting the ammonium formate and acetonitrile, allowing trapping of the eluting peptides onto a Symmetry C18, 180μm x 20mm trapping cartridge (Waters). After 15 min washing of the trap column, peptides were separated using an in-line second dimension analytical separation performed on a 75μm ID x 200mm,1.7μm BEH130 C18, column (Waters) using a linear gradient of 5 to 40% B (buffer A = 0.1% formic acid in water, buffer B = 0.1% formic acid in acetonitrile) over 90 min with a wash to 85% B at a flow rate of 300 nl/min. All separations were automated and performed on-line to the mass spectrometer.

All mass spectrometry was performed using a Synapt Q-Tof mass spectrometer fitted with a nanolockspray source operating in MS^e^ mode (Waters, Manchester, UK). Data was acquired from 50 to 1990 *m/z* using alternate low and high collision energy (CE) scans. Low CE was 5V (Trap), 4V (Transfer) and elevated was 12-35V ramp (Trap), 10V (transfer). The lock mass Glu-fibrinopeptide, (M+2H)^+2^, *m/z* = 785.8426) was infused at a concentration of 100 fmol/μl at 250 nl/min and data acquired every 60 seconds.

### Data processing

ProteinLynx Global Server 2.3 was used to process each raw data file, to generate reduced charge state and deisotoped precursor and associated product ion mass lists. Each processed file was searched against a protein translation of the *C*. *trachomatis* L2/434/Bu (897 entries, Jan, 2008) and Human genome sequence (Mar, 2009) including the L2 plasmid sequence (8 entries, Jan, 2008) [19] and the internal standard alcohol dehydrogenase (*Saccharomyces cerevisiae*) using the PLGS database search algorithm IDENTITY^E^ (Waters). Search parameters were as follows: Precursor and product ion tolerance were 10 ppm and 15 ppm respectively. Data were also searched against a combined database of the *C*. *trachomatis* L2/434/BU genome and all Uniprot entries available for the human proteome. Peptides that were homologous between *C*. *trachomatis* and human were removed from the dataset using an in-house script. A false positive rate of 4% was applied. Data was further filtered for protein quantitation by only considering proteins that were identified in at least two replicates of the same developmental form. By using the replication of protein assignments across different injections, the false positive rate is minimized, since chemical noise is random in nature and does not end to replicate across injections [24]

### Data normalisation

Normalisation to obtain estimates of absolute concentration were calculated using the ‘Top3’ approach implemented within the software IDENTITY^E^ [25]. In brief, the average intensity of the ‘Top3’ most abundant peptides of the internal standard alcohol dehydrogenase was used to calculate a universal response factor (counts/ mol of protein). This response factor was applied to the average intensity of the ‘Top3’ most abundant peptides from all other assigned proteins to provide estimates of their absolute concentration on column. Each sample was normalized on a per bacterium basis. Based upon the assumption that one *omcB* gene (calculated as described above) is equivalent to one genome, and that one genome is equivalent to a single bacterium, the number of bacterial cells equivalent to the amount of digested protein lysate injected on column was determined and the number of molecules per bacterium calculated.

### Energy expended during protein synthesis

To a first approximation, the energy expended in synthesizing a specific protein within a cell was calculated as follows. 60 kcal/mol are required to extend a nascent polypeptide chain by a single amino acid [26]. This energy expenditure arises from (i) the synthesis of a codon encoding a specific amino acid, (ii) the charging of the tRNA by its synthetase with the cognate amino acid, and (iii) the incorporation of the amino acid into the nascent polypeptide chain. The required energy for this process is generated from the hydrolysis of 10 energy-rich bonds in the form of either ATP or GTP, each with an energy content of about ΔG = -6 kcal/mol. Therefore, the number of constituent amino acids of each protein obtained from the UniProt protein database was used to calculate the energy required to synthesise one molecule of each specific protein identified. Using the number of molecules per bacterium calculated for each protein, showing an increased concentration in EBs, the total amount of energy invested in synthesising a specific protein per bacterium was calculated.

## Results and Discussion

**Fig 1:**
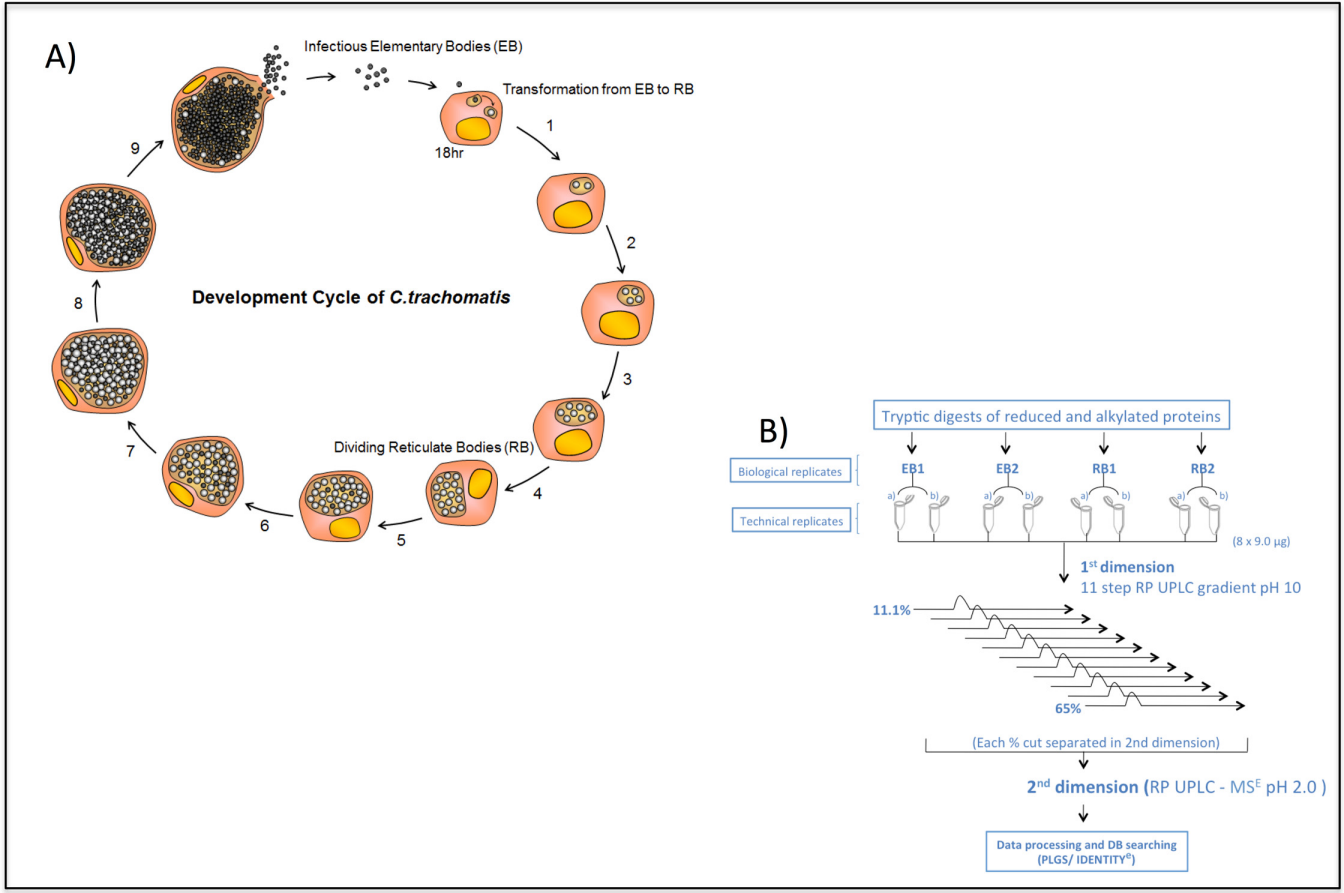
The *C*. *trachomatis* L2 developmental cycle (**A**) and the experimental workflow for the analysis of highly purified *C*. *trachomatis* L2 EBs and RBs using 2D-RP-RP-LC-MS^E^ label-free technology (**B**).

*C*. *trachomatis* L2 is our model system of choice for studying proteomics, genome analysis and chlamydial transformation. This bacterial isolate belongs to the lymphogranuloma ‘biovar’ from which strains cause invasive disease and they are thus not typical of urogenital chlamydia which are confined to the mucosal surfaces, but LGV isolates are ideal for laboratory analyses of basic biology in cell culture as they are fast growing and have a high particle to infectivity ratio. The specific strain we chose for this study is *C*. *trachomatis* L2/4343/Bu which has a high quality matching genome sequence [19]. It was subject to three plaque purifications to ensure clonality and all subsequent passages were subject to rigorous testing for mycoplasma. In our work we used BGMK cells as the eukaryotic host since these cells grow very evenly and have a distinctive morphology that allows ready identification of inclusions by phase contrast microscopy. The BGMK cells are clonal, easily infected and centrifugation is not required to initiate infection by *C*. *trachomatis* L2, which means the process is efficient. The bacteria are endocytosed into a cytoplasmic vesicle which is then subverted by microbial processes to become an ‘inclusion’. In BGMK cells the process of conversion from EB to dividing RB for *C*. *trachomatis* L2 occurs within inclusions and takes some 18hrs, inclusions are just visible by phase contrast microscopy, after this time the RBs rapidly divide by binary fission [12]. We have carefully calibrated this process and found that the earliest time it is possible to harvest and purify an homogenous population of RBs, is 15hrs post infection. This is just prior to the commencement of both bacterial cell division and chromosomal replication, our experimental design is shown in **Fig 1A**. Since asynchrony arises in the developmental cycle soon after this point, it is only possible to purify early RBs and mature EBs as distinct bacterial populations, within the constraints of the current technology. RBs and EBs were extracted from infected cells as previously described and then purified through two cycles of density gradient centrifugation followed by overlaying onto a discontinuous gradient. The purity of EBs and RBs was assessed by TEM as previously described [16, 27] and preparations were >99% pure for RBs and EBs. Yields of RBs were low at 2 mg for 18 x T175 flasks at moi of 3.0 when 95% of the cells are infected, we found it was not possible to obtain reasonable yields at lower moi, and above this threshold especially at moi = 10, EBs were cytotoxic in our experimental system [12].

### Proteome coverage

**Fig 2:**
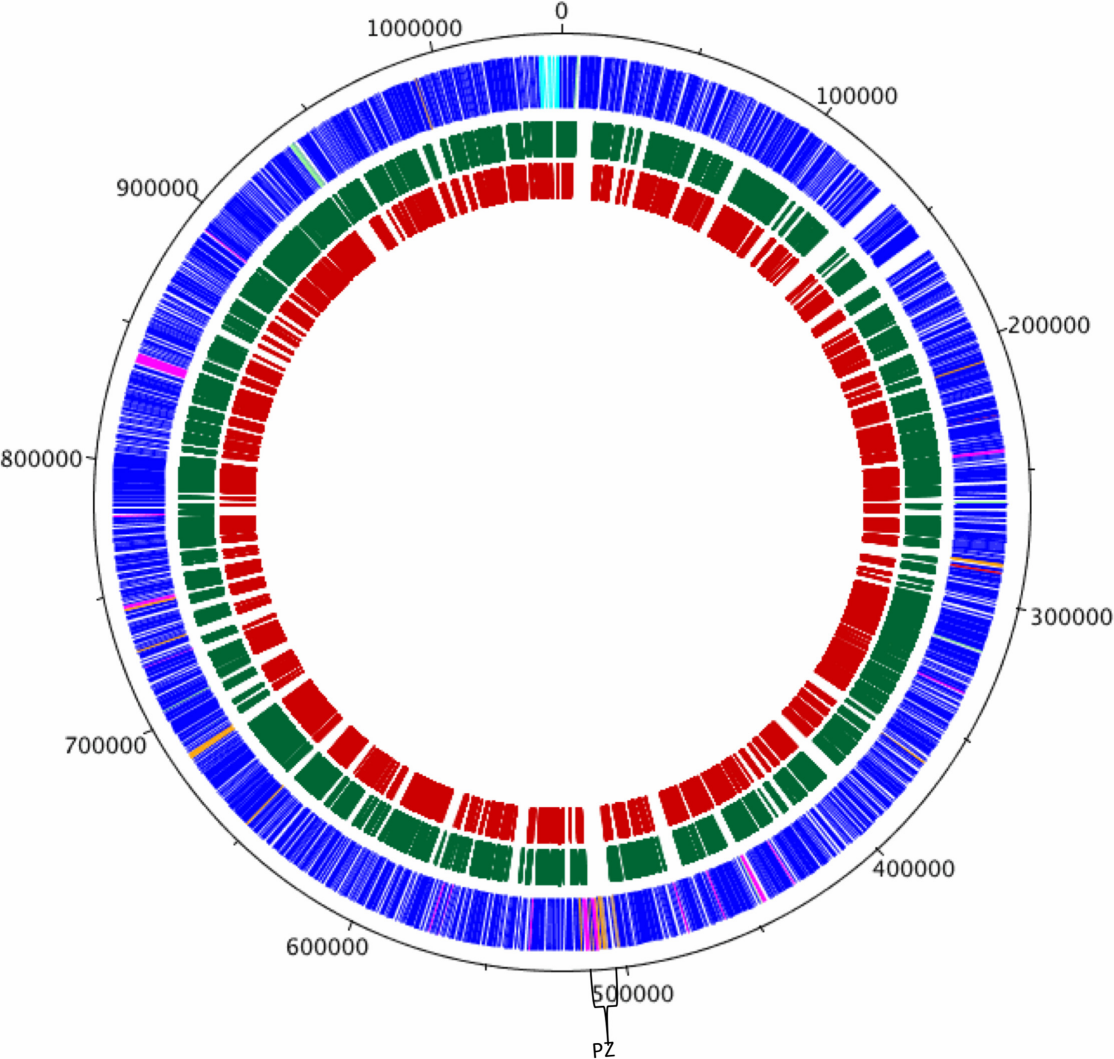
Circular chlamydial genome with peptide data mapped to CDSs. Circular representation of the *C*. *trachomatis* L2 chromosome and the mapping of peptides assigned from both EBs and RBs to their corresponding CDS. The outer scale shows the size in bp. The outer circle shows the positions of the CDS in a clockwise direction. The green and red circles indicate the CDSs of the peptides assigned in RBs and EBs, respectively. Using the published gene predictions for *C*. *trachomatis* strains UW-3 and Har-3, the strain L2 CDSs have been colour coded depending on whether the are: (blue) predicted and intact in all isolates; (pink) predicted and intact in L2 and UW-3; (green) predicted and intact in L2 and Har-13; (orange) defunct in L2, predicted and intact in Har-13 and UW-3; (red) unique to L2; (brown) defunct in all isolates. The region spanning the plasticity zone (PZ) is indicated.

Tryptic peptides prepared from EB and RB samples harvested at 15 h and 48 h post-infection, respectively, were analysed using a two-dimensional reverse phase UPLC-MS^E^ strategy (**Fig 1B**). Two biological replicates were prepared for each sample (2 x purified EB, 2 x purified RB). Two complete protein quantification profiles were obtained for each biological sample to give the identity and an estimate of the absolute amount of each protein within each sample. The mass spectrometry proteomics data have been deposited to the ProteomeXchange Consortium [27] via the PRIDE partner repository with the dataset identifier PXD003025. **Fig 2** highlights the comprehensive peptide coverage obtained for RBs and EBs across the chlamydial genome. Protein identification by this method was supported by an average of 46 peptides per protein (a minimum of 64% sequence coverage, (**S1D–S1K Fig**) while comparison of absolute protein estimates between technical replicates for protein lysates indicated an R^2^ value of 0.961 for EBs (**S1A Fig**) and 0.808 for RBs (**S1B Fig**), with mean % coefficients of variation between technical replicates, ranging from 12.9 to 16.9% across the dataset. Altogether, 562 proteins were identified and estimates of absolute amounts were collected for 489 proteins (**S1 Table**), representing ~62% and 54% of the total chlamydial proteome for EBs and RBs respectively. Our proteomic analyses were not designed to capture specifically exported proteins and, by definition, inclusion membrane proteins are excluded from this approach since we only sought to analyse two discrete bacterial populations, and not the membranes that bounded them. Nevertheless, 4 well characterized inclusion membrane and 7 candidate inclusion membrane proteins were detected. In every case, these proteins were more abundant in purified RBs. Since the extensive density gradient purification protocols (which are severely detrimental to the preservation of fragile membrane structures) were used to purify EBs and RBs, we conclude that these inclusion membrane proteins are synthesized *de-novo* and are ‘in transit’.

Overall proteome coverage compares very favorably with the most recent study where 54% and 42% coverage of the predicted EB and RB proteome of *C*. *trachomatis* L2 was catalogued, respectively [17]. Although we expected some similar biological observations to Saka et al., their experimental design was different and they cultured *C*. *trachomatis* using a different cell line (Hela cells) to that in our study (BGMK), they also attempted to purify inclusion membrane proteins and used a different infection strategy (multiplicity of infection).

### Most abundant proteins

**S2 Table** summarizes the data for the 15 most abundant proteins in RBs, whilst **S3 Table** shows the equivalent data for EBs. The dynamic expression range minimally spans three orders of magnitude from 0.96 to 1,428 fmol on column. In keeping with previous results [28, 29], the major outer membrane protein, MOMP, was found to be the most highly abundant component in EBs and the second most abundant in RBs. The hitherto hypothetical gene product CTL0847 was also amongst the most highly expressed components in *C*. *trachomatis*. The fact that highly abundant uncharacterized proteins were not uncovered previously underscores the generally poor correlation between mRNA and protein abundance data, as well as the power of the MS^E^ approach.

For EBs and RBs, protein abundance can also be expressed as estimates of molecules/bacterial cell (**S1, S2 and S3 Tables**). Thus, MOMP is present at approximately 2700 copies per bacterial cell, whilst proteins such as the conserved hypothetical protein CTL0455 and tetratricopeptide repeat protein, CTL0052 were present at fewer than 15 copies per cell. Proteins in the second category, that are also essential for cell viability may represent particularly attractive targets for antimicrobial drugs as relatively few copies need to be inactivated in order to block growth.

### The energy invested into making different classes of proteins

**Fig 3:**
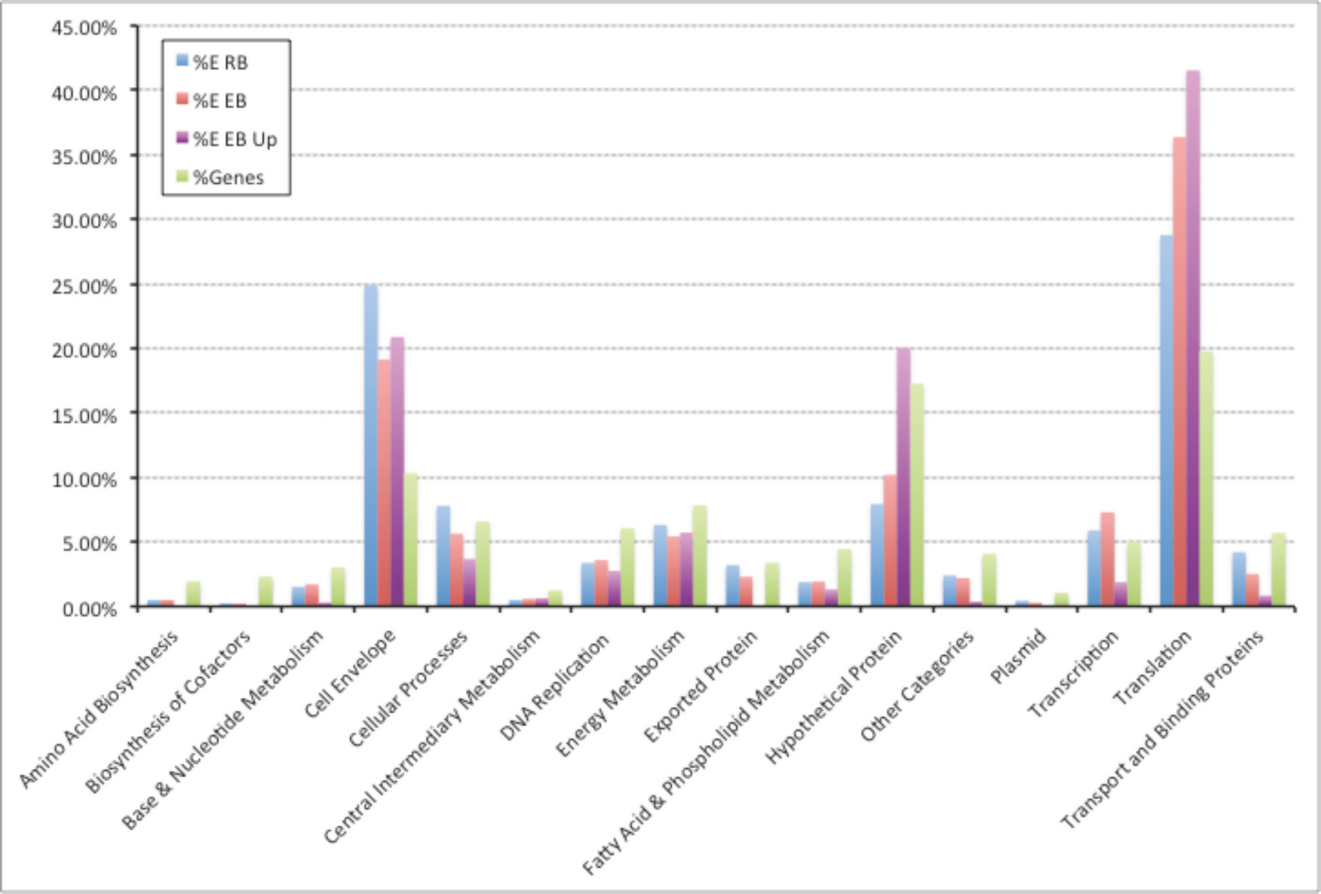
Estimated percentage energy investment in different functional classes of proteins for proteins present in 2+ EB replicates and/or 2+ RB replicates. Blue, RB proteome; Red, EB proteome; Purple, proteins up-regulated in EB; Green, percentage of proteome in functional class.

Comparison of the absolute quantification data for RBs and EBs suggests that protein levels were generally lower in EBs although certain proteins, e.g. integration host factor (CTL0519), acyl carrier protein (CTL0488) were present at higher levels. The absolute quantitation data has permitted estimates of the energy invested in the synthesis of proteins belonging to different functional categories. Our results indicate that *C*. *trachomatis* expends significant amounts of energy into maintaining the translation machinery and synthesizing proteins associated with cell envelope function, as well as hitherto hypothetical proteins (**Fig 3**). The majority of cell envelope related commitment was concerned with the synthesis of MOMP. We speculate that maintaining the translational components is critical during the initial stages of infection and hence are accumulated and maintained in EBs in anticipation of this event.

### Expression of cell wall enzymes in EBs and RBs

The presence of an essentially complete set of genes for peptidoglycan biosynthesis, together with the penicillin sensitivity of *C*. *trachomatis*, strongly suggests that the pathogen has the capacity to build a partial cell wall and the existence of such a peptidoglycan structure was recently proven using click chemistry [30]. Our UPLC-MS^E^ analysis detected the majority of enzymes involved in peptidoglycan synthesis which, in keeping with previous transcriptomic and more limited proteomic studies [14, 31, 32], were generally only expressed in significant amounts in the RB form (**S2 Fig**). The exception to this trend was muramidase, which showed 1.4-fold elevation in EBs. This suggests that peptidoglycan structures synthesized in the RB are actively degraded as the pathogen switches to its extracellular infectious form. Consistent with the proposed role of the cytoskeletal protein mreB (CTL0078) in cell division, we observed elevated levels in the early RB.

### Type III secretion

Since proteins exported from the cell are ‘lost’ to the pathogen they present a greater burden on the bacterial cell, as their amino acids cannot be recycled. Thus evolution of the secreted proteins and the apparatus to secrete them represents a critical step in intracellular adaptation. Given that chlamydia only have a very limited ‘window’ to exploit the host eukaryotic cell, we hypothesize that the process of evolution to an intracellular existence has ‘optimized’ the use specific use of extracellular proteins so that function of the chlamydial TTSS is rapid and highly efficient, especially in the early stage of infection.

The *Chlamydia* encode between 20 to 30 genes for structural proteins and chaperones of the TTSS [33, 34]. Our study has provided quantitative data for 15 structural proteins (**Table 1**), 4 chaperones and 25 effector proteins of the TTSS including CopD, CopB, CopN, Pkn5 and CADD. The expression levels of the predicted structural TTSS proteins, with the exception of SctW (CTL0344) showed reduced levels of expression in the late stage (48 h PI) of infection and in some cases, were only present in RBs at 15 h PI. Collectively, these data suggest an increased TTS capacity in RBs at 15 h PI that is reduced later in the developmental cycle, an observation consistent with the reported expression of the TTS-specific genes mid-cycle [35] and at a time when the intra-vacuolar environment is being heavily modified by predicted TTS substrates [36]. Relatedly, the predicted effector proteins, including IncA, IncE, IncG, IncC, and 7 additional predicted inclusion membrane proteins were also detected and, where quantitative data was available, showed decreased levels in EBs, indicating their secretion. The exception to this trend was the multi-cargo effector chaperone Mcsc, which was equally abundant in both RBs and EBs and the effector protein CADD (Chlamydia protein associating with death domains, CTL0874), which was more abundant in EBs and has previously been shown to be expressed late in the developmental cycle and to modulate host cell apoptosis [37].

**Table 1:** Structural proteins of the Type III secretion apparatus identified.

Locus	Protein	RB	EB	Location
CTL0345	SctV/LcrD/CdsV	252±132	65±20	IM
CTL0344	SctW/LcrE/CopN	54±11	42±18	Secreted
CTL0038	SctN/CdsN	91±11	45±18	CP:MA
CTL0041	SctQ/CdsQ	162±68	54±10	IM
CTL0825	SctR/CdsR	32±16	ND	IM
CTL0826	SctS/CdS	ND	ND	IM
CTL0826	ScT/CdsT	ND	ND	IM
CTL0036	SctU/CdsU	33±10	ND	IM
CTL0043	SctC/CdsC	260±125	75±12	OM
CTL0033	SctD/CdsD	924±441	341±7	IM
CTL0035	SctF/CdsF	ND	ND	Needle
CTL0822	SctJ/CdsJ	566±249	228±24	Link between IM & OM
CTL0824	SctL/CdsL	159±35	115±27	CP
CTL0841	CopB	73±8	31±5	HCM
CTL0842	CopD	134±21	76±12	HCM TL
CTL0238	LcrV	70±14	55±28	HCM TL

IM: inner membrane; OM: outer membrane; CP: cytoplasmic; MA: membrane associated; HCM: host cell membrane; TL: translocon component. Nomenclature adapted from Beeckman and Vanrompay, 2010 [38]

Whereas we observe a reduced capacity later in the developmental cycle, Saka et al.,[17] reported a marked absence of TTSS components in RB and proposed a reduced TTSS capacity, or a limited number of active TTSS apparatus in RBs. Notably, the absence of the C-ring components of the TTSS basal body, SctQ and the ATPase, SctN in RBs, led the authors to suggest substitutes for these components in the RB form. By contrast, our data indicate higher expression levels of the TTSS components in RBs with reduced expression in EBs, a trend that is consistent with the decrease of the TTS-like projections per bacterium, observed during the transition from RB to EB. Additionally, the C-ring components, SctQ and SctN were expressed at 162 ±68 and 91 ±11 molecules/cell in RBs, respectively, and 54 ±10 and 45 ±18 molecules/cell in EBs, respectively. To validate our data in the TTSS we compared expression levels by immunoblot of CT0847, a type III secretion structural protein associated with the needle tip component from *C*. *trachomatis* L2 in purified EBs and RBs. CT0847 is equivalent to CT584 in urogenital *C*. *trachomatis* and Cpn0803 in *C*.*pneumoniae*, it is one of the most highly abundant proteins (S1 Table). Our immunoblot data (**S3 Fig**) support our quantitative proteomics data and show a nearly equal abundance of this TTSS protein in both forms. We postulate that the expression of SctQ and SctN is ramped-up to pre-pack future EBs, and we observe expression of these TTS components at 15 h PI. Nonetheless, our data does show the expression of TTSS components in EBs, albeit at lower levels than RBs, supporting the hypothesis that EBs are pre-loaded with TTSSs [33].

### Energy metabolism

**Fig 4:**
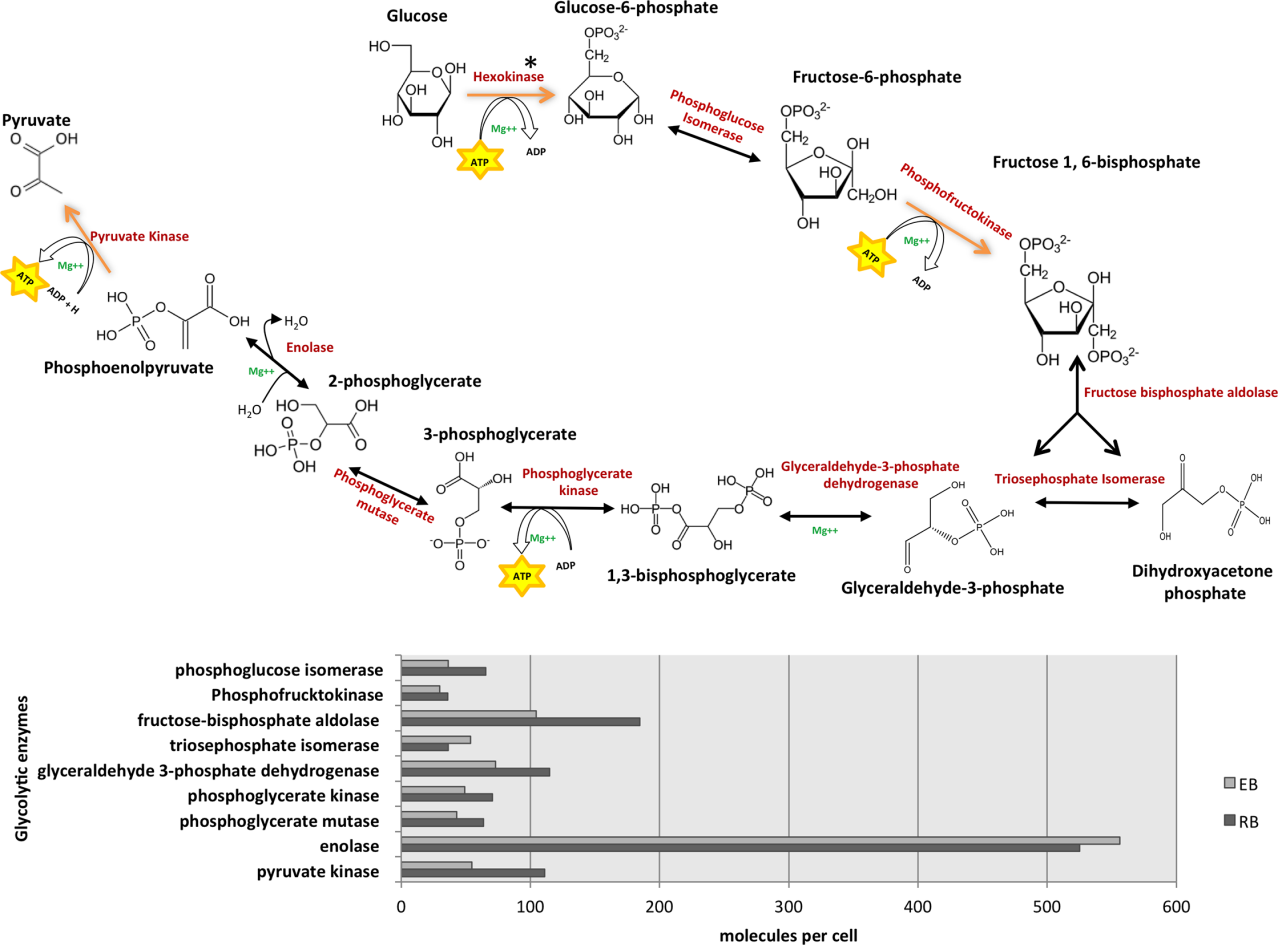
Representation of the glycolytic pathway in *C*. *trachomatis* L2 (A) and associated expression levels of each glycolytic enzyme from 15 to 48 h PI (B). *There is no hexokinase homolog in the *C*. *trachomatis* L2 genome.

Until recently, the dogma has been that EBs are metabolically dormant. However, we identified all the chlamydia-encoded enzymes for glycolysis, tricarboxylic acid cycle and pentose phosphate pathway in both RBs and EBs. This suggests that chlamydiae are capable of ATP synthesis via glucose catabolism throughout their developmental cycle. Omsland et al. (2012) [18] using axenic culture have shown that both EBs and RBs are able to perform *de novo* protein synthesis and generate ATP. They also showed that EBs preferentially required glucose-6-phosphate and RBs showed further enhanced activity in the presence of ATP. In keeping with this, the quantitative measurements in our study indicate that these glucose metabolism enzymes, including the ADP/ATP translocase, are at their most abundant in the RB form (15 h PI), and show a general trend for decreased levels of expression in EBs (48 h PI). A summary of the expression levels of these glycolytic enzymes is shown in **Fig 4**. Despite the higher levels of these enzymes in RBs, the abundance of these enzymes in EBs is significant, confirming that chlamydiae have the capability to generate ATP via substrate-level phosphorylation throughout their developmental cycle, an observation that is consistent with previous RT-PCR results, which showed maximal expression of the genes PK, GAPDH, PGK and ZWF in RBs [39]. We have calculated to a first approximation, the functional categories where energy is expended in synthesizing proteins. Since EBs are metabolically active and also maintain a protein translation capability, proteins required for extracellular survival or the initial stages of infection can be synthesized on demand.

### Gaps in the proteome coverage

The major region of variation between coding sequences of the different chlamydial strains and species is the region termed the plasticity zone (PZ). This variant region is principally attributed to the loss of the cytotoxin gene(s), which have almost entirely been deleted from *C*. *trachomatis* L2 leaving two remnants CTL0420 and CTL0421 [40]; and 4 encoded phospholipase genes, CTL0409, CTL0411, CTL0413 and CTL0414. However, although CTL0409 and CTL0414 have acquired multiple frameshift mutations and deletions, CTL0411 and CTL0413 appear intact in UW-3 (serovar D), Har-13 (serovar A) and L2. Mapping of the identified peptides from RBs and EBs to the L2 genome (**Fig 2**), there is a striking absence of peptides detected across the PZ, although proteins encoded by the *trpRBA* operon, potentially linked with genital and ocular tropism were detected [41]. This absence would be expected for the predicted pseudogenes (**S4 Table**) and validates their assignment to this category. However, the clear and notable absence of peptides mapping to other predicted ‘functional’ genes able to encode proteins, such as the phospholipase genes, CTL0411 and CTL0413, spanning this same region, does raise the question whether these genes are expressed or whether they too are non-coding either at the level of transcription or translation.

Of the proteins not identified in this study, of particular note are hypothetical proteins, or proteins of unknown function, representing >10% of the predicted *C*. *trachomatis* L2 genome (**S4 Fig**). By comparison the remaining unidentified proteins were fairly evenly distributed across the remaining 13 functional categories. Interestingly, in a previous study focused on improving pseudogene assignment, using 11 genomes from 4 bacterial genera, the number of pseudogenes ranged from 27 in *Staphylococcus aureus* MW2 to 337 in *Yersinia pestis* CO92. Over half of these pseudogenes identified were previously annotated as ‘hypothetical’ [42]. Considering the high representation of ‘hypotheticals’ within this dataset, we speculate that these could also represent unassigned pseudogenes; or are they characteristically atypical preventing their detection; or are they simply not expressed under the conditions of measurement? Whatever the reason for the gaps in the proteome coverage, it is clear that accurate prediction of pseudogenes is required in defining our understanding of what represents a complete proteome.

### Concluding remarks

Proteome-wide determination of absolute protein quantities is a challenge of broad biological importance. In this study, we have determined and compared the levels of hundreds of proteins in the EB and earliest RB forms of *C*. *trachomatis*. Such data provides insights into the steady-state levels of proteins and hence cellular priorities for protein synthesis. It also allows exploration of structural, functional and genetic correlates of protein abundance (amino acid composition, pI, gene essentiality), and is an important prerequisite for predictive mathematical modeling of cellular processes. These high quality data are reproducible, robust and reliable for high abundance proteins. However, the subtleties of chlamydial gene regulation are likely to lie within the proteins of low abundance (<10 copies per cell). In pathogenic microbes these are of specific interest as they may be of use in the selection of targets for the development of new antimicrobial agents. It is likely that there is a greater variation in the copy numbers of low abundance proteins as feedback control of low concentrations is notoriously difficult, and easily leads to overshooting and random oscillations [43]. The availability of accurate quantitative proteomics data, indicating levels of protein/enzymes in RBs and EBs is a fundamental resource and will be invaluable for planning future experiments to manipulate the genome to investigate gene regulation and function.

## Supporting Information

S1 Fig Comparison of the absolute protein abundance measurements obtained from the analysis of two technical replicates of EBs (S1a) and RBs (S1b) from C. trachomatis L2 using 2D-LC-MSE. Protein abundance from replicate 1 and 2 are represented on a log2 scale. R^2^ = correlation coefficient.
(ZIP)

S2 Fig A schematic representation of the proposed chlamydial peptidoglycan biosynthesis pathway and related genes. The precursor, UDP-MurNAc pentapeptide is synthesized in the cytoplasm by six enzymes (MurA to Mur F). This precursor is subsequently transferred to the lipid carrier undecaprenyl phosphate catalyzed by MraY to form the first membrane bound intermediate, Lipid I. Catalysed by MurG, Lipid II is synthesized by the addition of UDP-GlcNAc to Lipid I, followed by translocation into the peptidoglycan structure. The table indicates those peptidoglycan biosynthetic enzymes expressed in *C*. *trachomatis* L2.
(TIF)

S3 Fig Immunoblot detection of the highly abundant proteins MOMP and the protein encoded by CTL0847. SDS–PAGE (A) and western blot analyses (B and C) of gradient–purified EBs and RBs. EBs and RBs were loaded in the gel tracks as indicated. In panel B the MOMP protein (~40kDa) was detected by monoclonal antibody 6Ciii and in panel C the protein encoded by CTL0847 was detected with a polyclonal mouse antiserum specific for this protein. The migration of the molecular weight markers are as indicated.
(TIF)

S4 Fig *C*. *trachomatis* L2 proteins not yet identified in this study, distributed according to functional category. (TIF)

S1 Table Main data table. (XLSX)

S2 Table 15 Most abundant proteins in RB. (PDF)

S3 Table 15 most abundant proteins in EB. (PDF)

S4 Table Pseudogenes in *C. trachomatis* L2/434/Bu identified by whole genome comparison with *C. trachomatis* strains UW-3 (serovar D) and Har-13 (serovar A). (PDF)
