# Supplementary figures and images for: Quantitative Proteomics of the Infectious and Replicative Forms of Chlamydia trachomatis
Source: PLoS One. 2016 Feb 12;11(2):e0149011. doi: 10.1371/journal.pone.0149011 (PMC4752267; doi:10.1371/journal.pone.0149011)

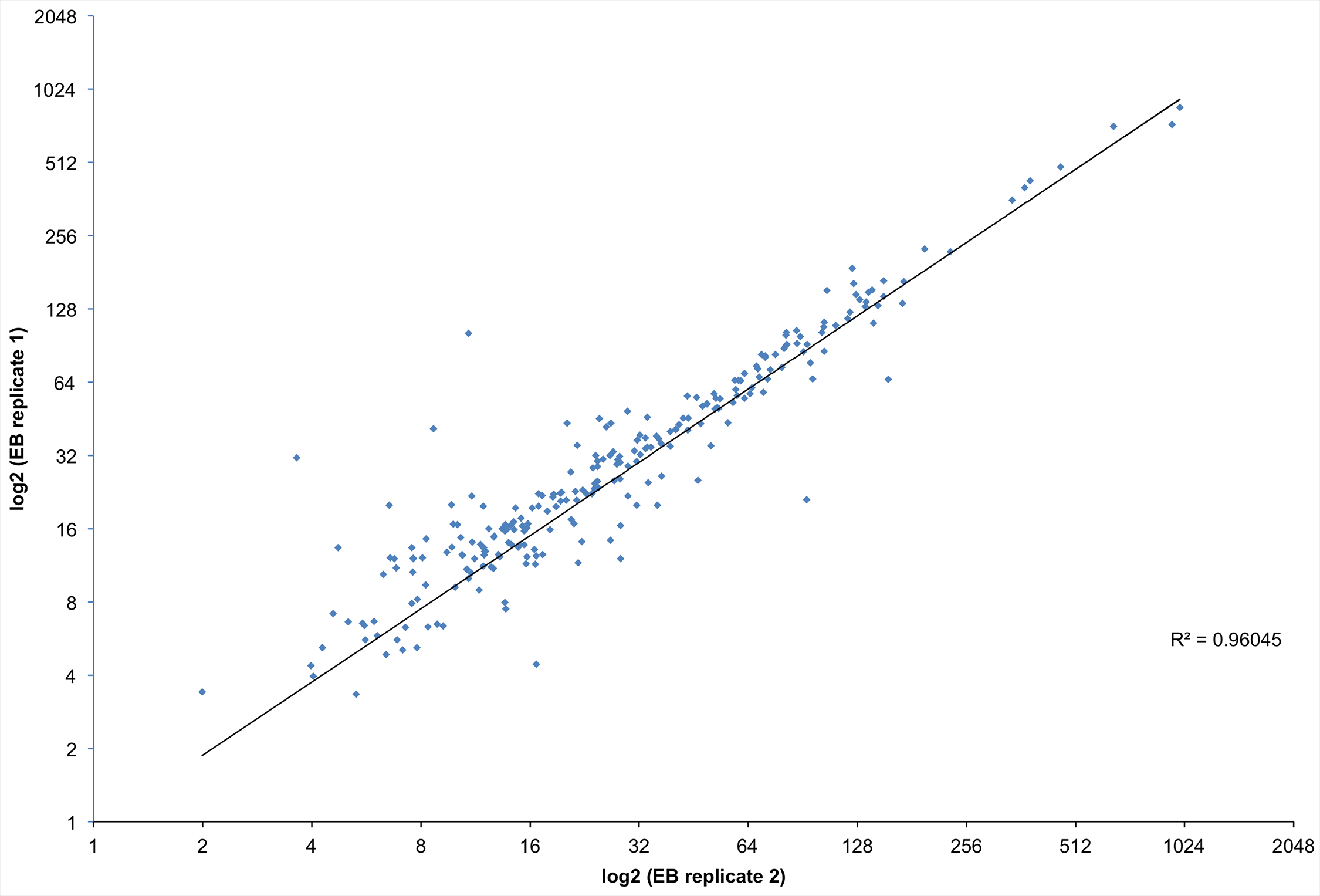

Supplement: S1 Fig — Protein abundance from replicate 1 and 2 are represented on a log2 scale. R2 = correlation coefficient. (ZIP) [file pone.0149011.s001.zip › Skipp.FigS1a.tif]

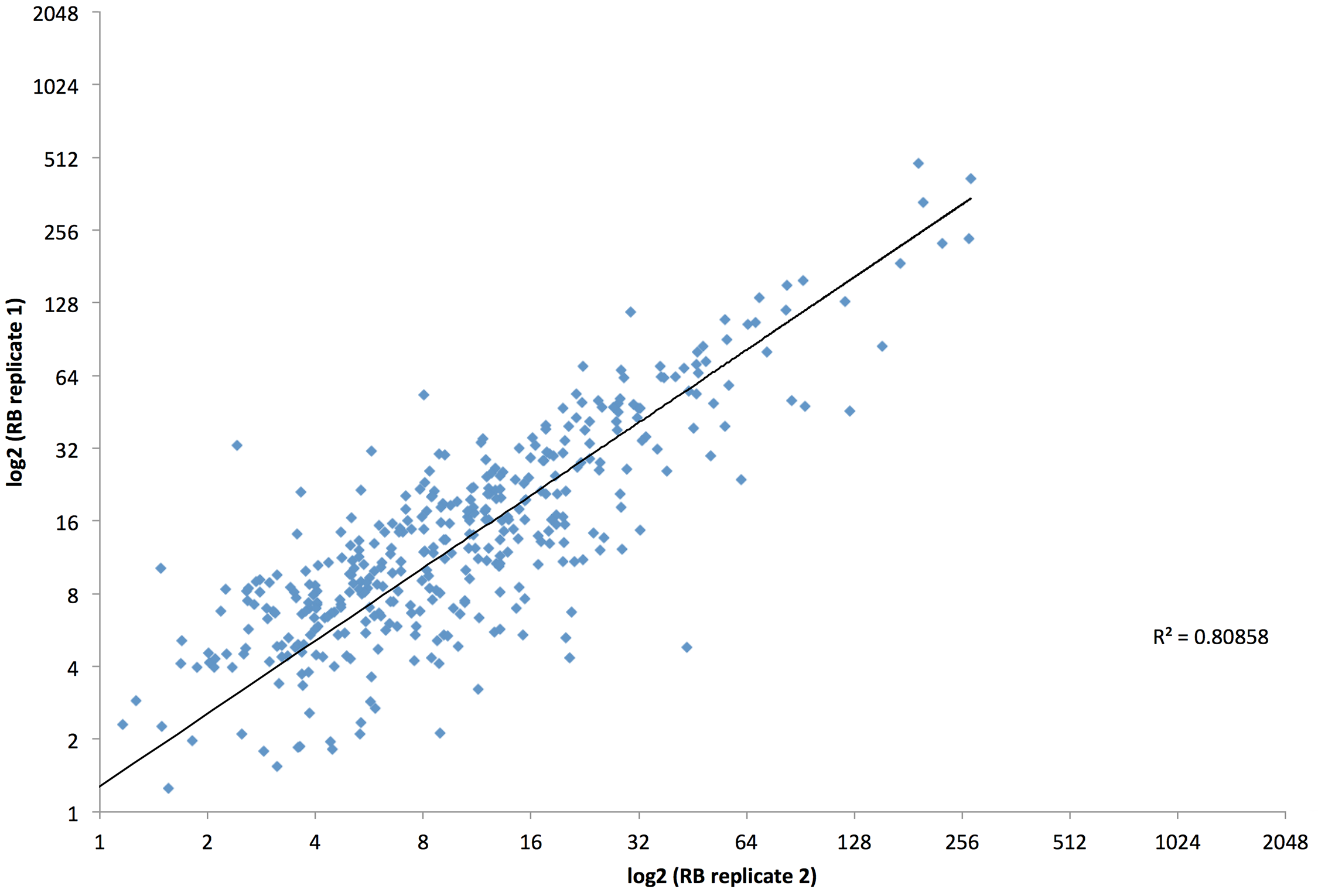

Supplement: S1 Fig — Protein abundance from replicate 1 and 2 are represented on a log2 scale. R2 = correlation coefficient. (ZIP) [file pone.0149011.s001.zip › Skipp.FigS1b.tiff]

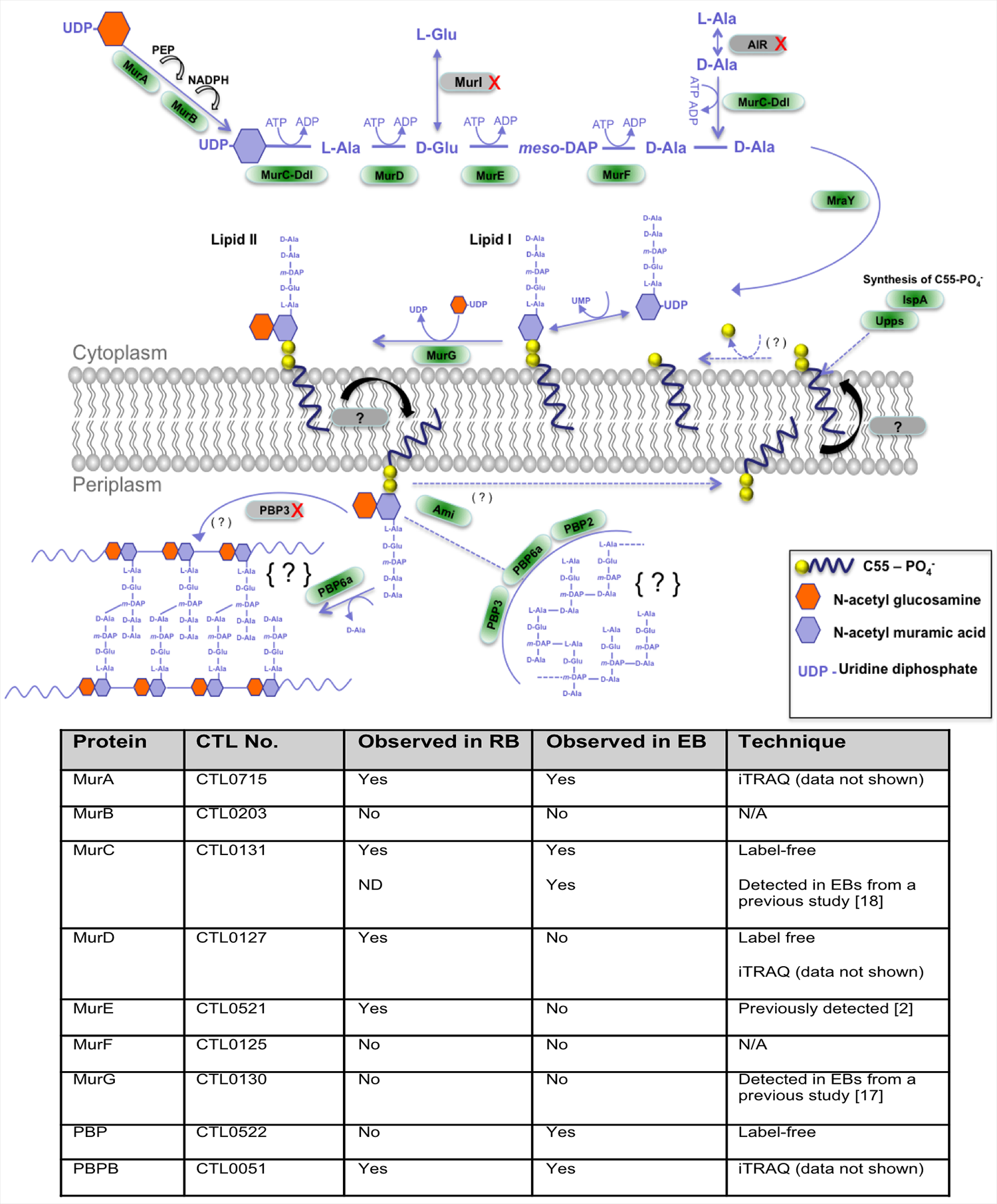

Supplement: S2 Fig — The precursor, UDP-MurNAc pentapeptide is synthesized in the cytoplasm by six enzymes (MurA to Mur F). This precursor is subsequently transferred to the lipid carrier undecaprenyl phosphate catalyzed by MraY to form the first membrane bound intermediate, Lipid I. Catalysed by MurG, Lipid II is synthesized by the addition of UDP-GlcNAc to Lipid I, followed by translocation into the peptidoglycan structure. The table indicates those peptidoglycan biosynthetic enzymes expressed in C. trachomatis L2. (TIF) [file pone.0149011.s002.tif]

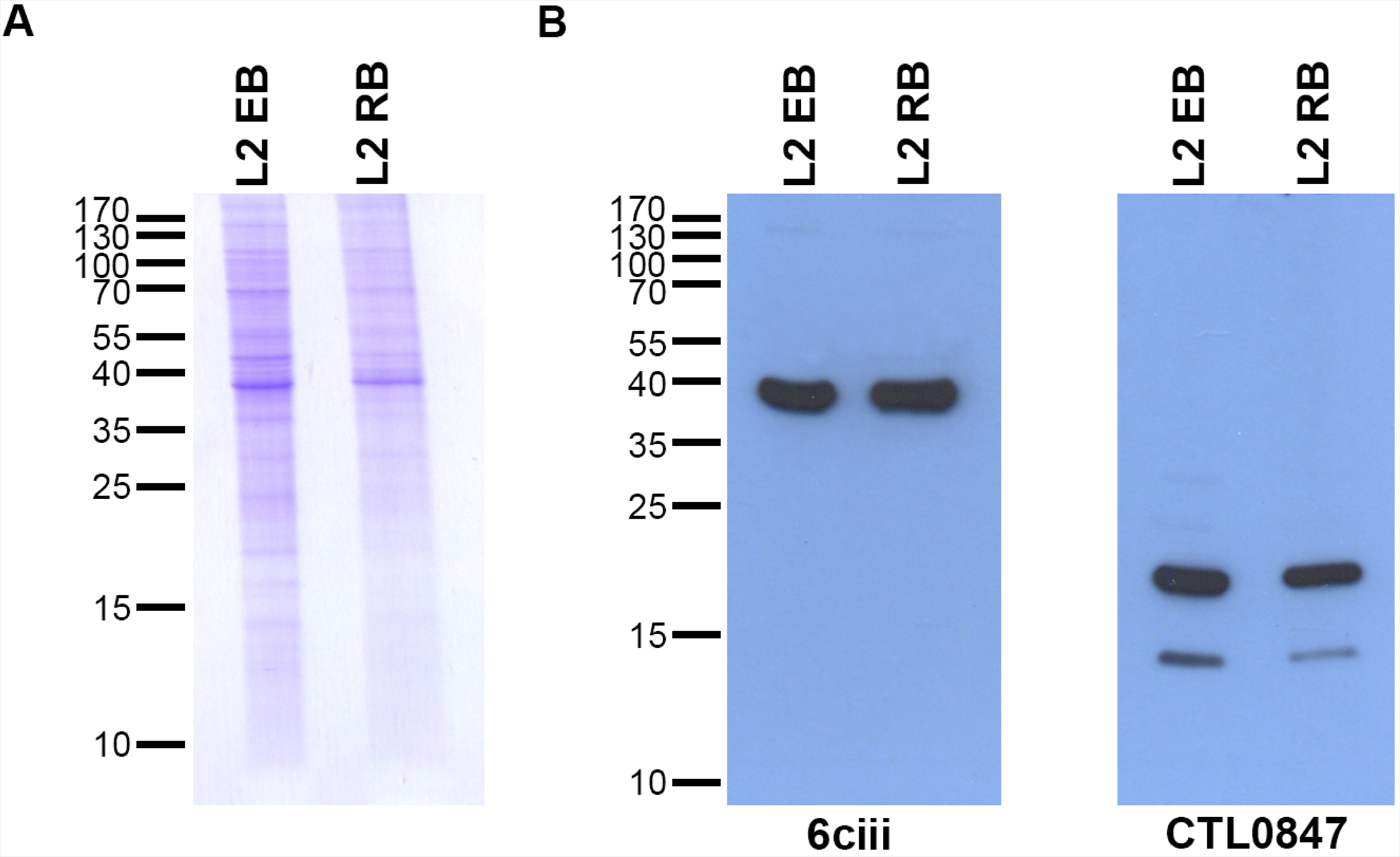

Supplement: S3 Fig — SDS–PAGE (A) and western blot analyses (B and C) of gradient–purified EBs and RBs. EBs and RBs were loaded in the gel tracks as indicated. In panel B the MOMP protein (~40kDa) was detected by monoclonal antibody 6Ciii and in panel C the protein encoded by CTL0847 was detected with a polyclonal mouse antiserum specific for this protein. The migration of the molecular weight markers are as indicated. (TIF) [file pone.0149011.s003.tif]

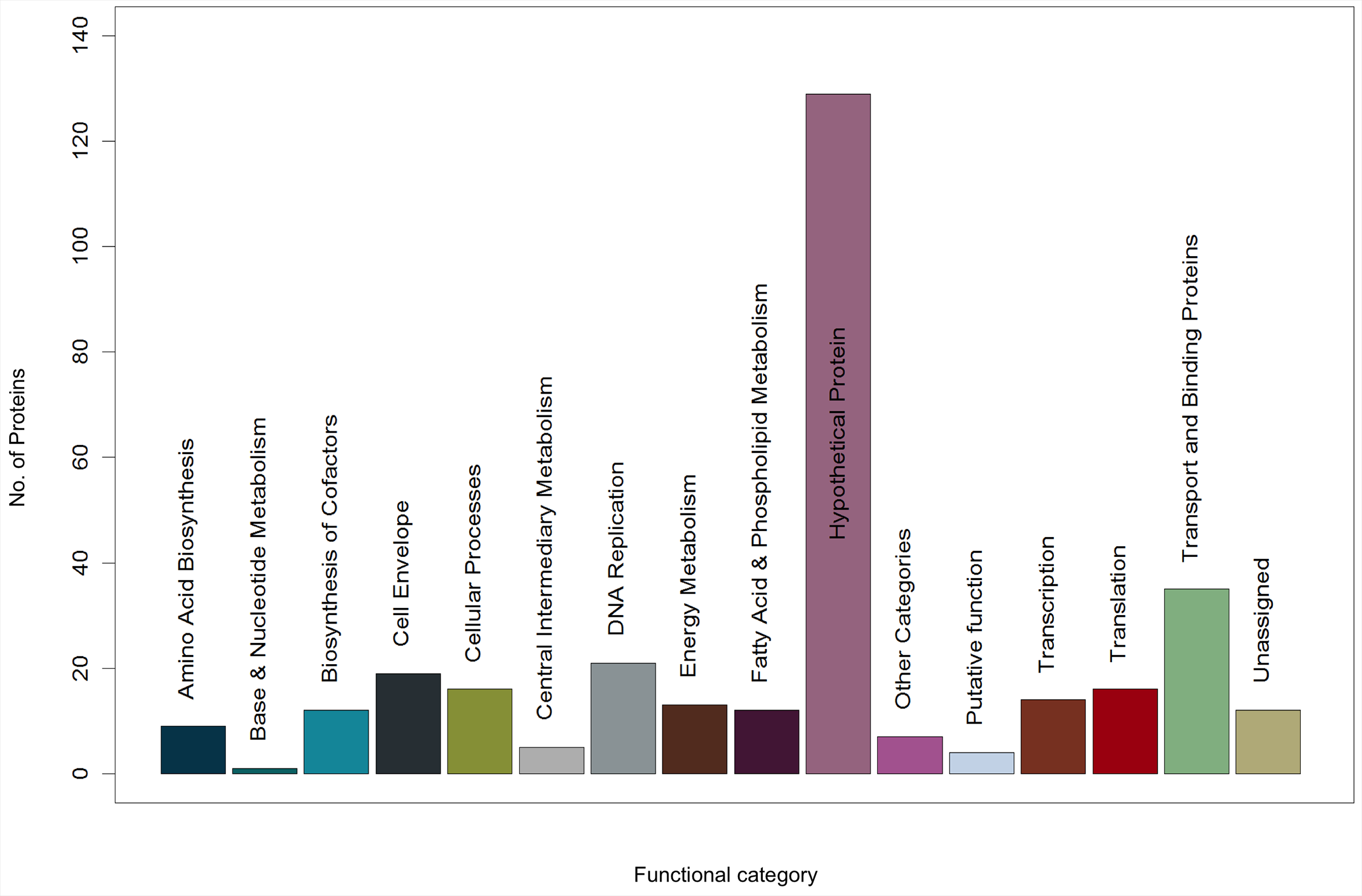

Supplement: S4 Fig — (TIF) [file pone.0149011.s004.tif]
