# Supplementary material for: Quantitative Proteomics of the Infectious and Replicative Forms of Chlamydia trachomatis
Source: PLoS One. 2016 Feb 12;11(2):e0149011. doi: 10.1371/journal.pone.0149011 (PMC4752267; doi:10.1371/journal.pone.0149011)
Supplement: S2 Table — (PDF) [file pone.0149011.s006.pdf]

Table S2. Top 15 most abundant proteins in RB

UniProt accession	Locus	Gene name	Protein description	RB (molecules/cell)	EB (molecules/cell)	EB/RB ratio	Functional Category
B0B7N8	CTL0574	tufA	translation elongation factor Tu	2619	2156	0.8	Translation
B0B8Q7	CTL0050	ompA	major outer membrane protein	2041	2728	1.3	Cell Envelope
B0B8B5	CTL0803	mip	peptidyl-prolyl cis-trans isomerase	1956	1292	0.6	Other. Categories
B0B9L8	CTL0365	hsp60_1	chaperonin GroEL	1871	1300	0.6	Translation
B0B815	CTL0702	omcB	60kD cysteine-rich outer membrane protein	1704	518	0.3	Cell Envelope
B0B8J8	CTL0887		putative exported protein	1464	840	0.5	Exported protein
B0B7W6	CTL0652	dnaK	chaperone protein	1424	1660	1.1	Translation
B0B9X4	CTL0476		candidate inclusion membrane protein	968	186	0.1	Cell envelope
B0B9H5	CTL0323		ABC transport protein_ solute binding component	950	379	0.3	Transport and binding Proteins
B0B8P0	CTL0033		phosphopeptide binding protein (predicted to be a TTSS protein)	924	341	0.3	Cellular Processes
B0B8F8	CTL0847		conserved hypothetical protein	914	1145	1.2	hypothetical protein
B0B9H3	CTL0321		ADP_ATP carrier protein	828	252	0.3	Energy Metabolism
B0B952	CTL0195	htrA	serine protease	774	399	0.5	Translation
B0B940	CTL0183	pmpD	polymorphic outer membrane protein	685	361	0.5	Cell envelope
B0B9A3	CTL0250	pmpG	polymorphic outer membrane protein	637	154	0.2	Cell envelope
