# Supplementary material for: Quantitative Proteomics of the Infectious and Replicative Forms of Chlamydia trachomatis
Source: PLoS One. 2016 Feb 12;11(2):e0149011. doi: 10.1371/journal.pone.0149011 (PMC4752267; doi:10.1371/journal.pone.0149011)
Supplement: S3 Table — (PDF) [file pone.0149011.s007.pdf]

Table S3. Top 15 most abundant proteins in EB

UniProt accession	Locus	Gene name	Protein description	RB (molecules/cell)	EB (molecules/cell)	EB/RB ratio	Functional Category
B0B8Q7	CTL0050	ompA	major outer membrane protein	2041	2728	1.3	Cell Envelope
B0B7N8	CTL0574	tufA	translation elongation factor Tu	2619	2156	0.8	Translation
B0B7W6	CTL0652	dnaK	chaperone protein	1424	1660	1.1	Translation
B0B9L8	CTL0365	hsp60_1	chaperonin GroEL	1871	1300	0.6	Translation
B0B8B5	CTL0803	mip	peptidyl-prolyl cis-trans isomerase	1956	1292	0.6	Other. Categories
B0B8F8	CTL0847		conserved hypothetical protein	914	1145	1.2	hypothetical protein
B0B7N2	CTL0568	rplL	LSU ribosomal protein L12P (L7/L12)	626	1006	1.6	Translation
B0B8J8	CTL0887		putative exported protein	1464	840	0.5	Exported protein
B0B8I5	CTL0874		conserved hypothetical protein	461	807	1.7	hypothetical protein
B0B7F2	CTL0488	acpP	acyl carrier protein	502	662	1.3	Fatty Acid & Phospholipid Metabolism
B0B8Q5	CTL0048	tsf	translation elongation factor TS	453	584	1.2	Translation
B0B8G1	CTL0850	eno	enolase	525	556	1.0	Energy Metabolism
B0B815	CTL0702	omcB	60kD cysteine-rich outer membrane protein	1704	518	0.3	Cell Envelope
B0B9L9	CTL0366	groES	10 kDa chaperonin GroES	622	475	0.7	Translation
B0B7I3	CTL0519	ihfA	integration host factor alpha-subunit	204	472	2.3	DNA Replication
