# Supplementary material for: Quantitative Proteomics of the Infectious and Replicative Forms of Chlamydia trachomatis
Source: PLoS One. 2016 Feb 12;11(2):e0149011. doi: 10.1371/journal.pone.0149011 (PMC4752267; doi:10.1371/journal.pone.0149011)
Supplement: S4 Table — (PDF) [file pone.0149011.s008.pdf]

L2 *locus*	Protein description
CTL0161	Conserved hypothetical protein
CTL0228	Fumarate hydratase (FumC)
CTL0292	Conserved hypothetical protein
CTL0409	Phospholipase D protein
CTL0414	Phospholipase D protein
CTL0415	Conserved hypothetical protein
CTL0418	Putative membrane protein
CTL0420	Cytotoxin (adherence)
CTL0421	Cytotoxin (adherence)
CTL0426A	Conserved hypothetical protein
CTL0552	Putative integral membrane protein
CTL0578	Conserved hypothetical protein
CTL0612	Inner membrane protein
CTL0627	Pyruvoyl-dependent arginine decarboxylase
CTL0856	Succinate dehydrogenase (sdhC)
